# Hesperidin Functions as an Ergogenic Aid by Increasing Endothelial Function and Decreasing Exercise-Induced Oxidative Stress and Inflammation, Thereby Contributing to Improved Exercise Performance

**DOI:** 10.3390/nu14142955

**Published:** 2022-07-19

**Authors:** Maria Imperatrice, Iris Cuijpers, Freddy J. Troost, Mireille M. J. P. E. Sthijns

**Affiliations:** 1 BioActor BV, Gaetano Martinolaan 50, 6229 GS Maastricht, The Netherlands; 2 Food Innovation and Health, Department of Human Biology, Maastricht University, 6200 MD Maastricht, The Netherlands; f.troost@maastrichtuniversity.nl (F.J.T.); mireille.sthijns@maastrichtuniversity.nl (M.M.J.P.E.S.)

**Keywords:** hesperidin, citrus flavanones, polyphenols, antioxidant, physical activity, exercise performance, ergogenic aids, endothelial dysfunction

## Abstract

The regulation of blood flow to peripheral muscles is crucial for proper skeletal muscle functioning and exercise performance. During exercise, increased mitochondrial oxidative phosphorylation leads to increased electron leakage and consequently induces an increase in ROS formation, contributing to DNA, lipid, and protein damage. Moreover, exercise may increase blood- and intramuscular inflammatory factors leading to a deterioration in endurance performance. The aim of this review is to investigate the potential mechanisms through which the polyphenol hesperidin could lead to enhanced exercise performance, namely improved endothelial function, reduced exercise-induced oxidative stress, and inflammation. We selected in vivo RCTs, animal studies, and in vitro studies in which hesperidin, its aglycone form hesperetin, hesperetin-metabolites, or orange juice are supplemented at any dosage and where the parameters related to endothelial function, oxidative stress, and/or inflammation have been measured. The results collected in this review show that hesperidin improves endothelial function (via increased NO availability), inhibits ROS production, decreases production and plasma levels of pro-inflammatory markers, and improves anaerobic exercise outcomes (e.g., power, speed, energy). For elite and recreational athletes, hesperidin could be used as an ergogenic aid to enhance muscle recovery between training sessions, optimize oxygen and nutrient supplies to the muscles, and improve anaerobic performance.

## 1. Introduction

During exercise, skeletal muscle cells convert biological fuel (e.g., lipids, carbohydrates) into mechanical force to allow muscle contraction and therefore movement. The energy required for this motion is largely provided by the breakdown of adenosine triphosphate (ATP). Intramuscular stores of ATP can sustain only a short period of muscle activity. Therefore, ATP needs to be generated by anaerobic glycolysis and oxidative phosphorylation [1]. During exercise bouts lasting several minutes to hours, mitochondrial oxidative phosphorylation is responsible for almost all the ATP generated for the contracting skeletal muscles. This process is critically dependent on the respiratory and cardiovascular systems to ensure an adequate oxygen supply [2].

Blood flow is the main regulator of the skeletal muscles’ oxygen supply. Skeletal muscle contains a dense capillary network that serves to deliver oxygen and nutrients and remove waste products from the skeletal muscle cells [3]. To ensure adequate muscle oxygenation, blood flow increases during exercise capacity (i.e., capillaries’ numbers and diameters) [4,5].

The endothelium plays a major role in the regulation of blood flow to peripheral muscles and is crucial for muscle perfusion [6]. The vascular wall is composed of a monolayer of specialized cells, the endothelial cells, which form the interface between the underlying smooth muscle cells and the vascular lumen [7]. Endothelial cells regulate vascular permeability and maintain vascular tone [8]. Normal arterial function requires a balance between vasodilation and vasoconstriction, which is important for regulating blood flow and vascular tone during rest and exercise [9,10]. Nitric oxide (NO) is a strong vasodilatory and anti-inflammatory signalling molecule, that regulates vascular tone [7]. The release of NO by endothelial cells causes the dilation of an artery, which leads to an increase in blood flow. On the other hand, vasoconstriction is induced by the release of endothelin-1 (ET-1). Endothelial dysfunction (ED) can lead to a reduced NO availability or an increased ET-1 synthesis, release, or activity [7,11]. In addition, hydrogen peroxide (H_2_O_2_) might be an important factor in the regulation of vascular tone by functioning as an endothelium-derived hyperpolarizing factor (EDHF) leading to vasoconstriction [12]. The main mechanisms underlying the pathophysiology of ED are increased reactive oxygen species (ROS), inflammation and diminished NO production, and bioavailability [13].

Investigating the link between an individual’s endothelial function and skeletal muscle function is of great interest in the field of exercise physiology [14]. Oxygen delivery and the related mitochondrial capacity of the muscles are regarded as the primary limiting factors for endurance performance [15]. When muscle mass is overperfused during exercise, it has an extremely high capacity for consuming oxygen [15]. Therefore, improved muscle perfusion during exercise via vascular endothelial function can positively impact endurance exercise performance.

### 1.1. Excessive Production of ROS Results in Decreased Force Output and Decreased NO Availability

Skeletal muscle tissue contraction, which induces a higher oxygen demand, could induce an increased formation of ROS as a result of the increased mitochondrial activity. This can lead to incomplete oxidative phosphorylation during exercise. Short-term increased ROS formation during physical activity, if not excessive, has shown to be important for exercise-induced adaptations including enhanced mitochondrial biogenesis, cardiovascular adaptations, as well as the regulation of contractile force [16,17]. ROS induces redox-sensitive signalling pathways involving redox-sensitive kinases, phosphatases, and the transcription factor nuclear factor-κB leading to an induced skeletal muscle adaptation [16]. The harmful effects of excessive ROS formation can be counteracted by the endogenous antioxidant system comprising superoxide dismutase (SOD), catalase (CAT), reduced glutathione (GSH), and glutathione peroxidase (GPx). However, during long-term, high-intensity endurance exercise, the continuous ROS production may exceed the capacity of the cellular defence system leading to damage to DNA, lipids (lipid peroxidation), or protein in the muscles [18]. Furthermore, ROS generated during exercise modulates muscle contraction signalling pathways; low levels of ROS stimulate force output, whereas high levels attenuate this [19,20].

A possible trigger of ROS production in vascular cells could be the increased blood flow during exercise, thereby increasing the shear stress [21]. There is accumulating evidence suggesting that in ageing and certain disease states such as hypertension, atherosclerosis, and heart failure, there might be an excessive formation of ROS in response to exercise resulting in decreased NO availability through reaction of NO with superoxides [22,23,24]. Via a similar mechanism, the overproduction of ROS during high-intensity exercise (in healthy individuals) leads to a decline in NO availability, whereas supplementation with antioxidants (e.g., hesperidin) can reverse these adverse effects [25]. In this way, the overproduction of ROS is linked to impaired vascular homeostasis and ED.

### 1.2. Detrimental Effects of Post-Exercise Inflammation on Endurance Performance and Endothelial Function

The immune system plays a key role not only in protecting our bodies from invading microorganisms and disease prevention but also in wound-healing processes [26] and tissue-remodelling mechanisms [27]. Macrophages are specialized cells affecting inflammation and the healing response to acute injury [28]. When it comes to exercise immunity, macrophages play a key role in skeletal muscle regeneration [29]. Despite the fact that the exact macrophage-mediated signalling of inflammation and muscle regeneration is not yet fully understood, several cytokines, including tumour necrosis factor-alpha (TNFα), interferon-gamma (IFNγ), interleukin 6 (IL-6), and interleukin 10 (IL-10), appear to play key roles in the muscle regeneration process [30,31,32].

Although the exercise-induced inflammatory response is important to stimulate muscle adaptations [33,34,35], the post-exercise recovery period is equally critical in providing sufficient time for metabolic and structural adaptations to occur within skeletal muscle, e.g., skeletal muscle hypertrophy [30] and exercise-induced angiogenesis [36]. Unaccustomed exercise (in type, intensity, and duration of training), especially if it requires eccentric (muscle-lengthening) contractions, frequently leads to exercise-induced muscle damage (EIMD) [26]. EIMD is linked to an increase in inflammatory markers both within the injured muscle and the blood, the increased appearance of muscle proteins in the blood, and the delayed onset of muscle soreness (DOMS) [26,37,38]. The acute inflammatory response following EIMD is characterized by increased levels of circulatory and intramuscular inflammatory markers such as C-reactive protein (CRP) and cytokines (e.g., TNF-α and IL-6) [39,40,41].

Without adequate post-exercise/competition recovery periods, an excessive inflammatory response could lead to impaired muscle contractions and force production [30,42]. Moreover, post-exercise inflammation can inhibit the recovery of muscle function, thereby negatively impacting short-term recovery [43,44]. Therefore, if the highly demanding training schedule of professional athletes is not tempered with periods of rest and recovery, a short-term performance decrement can be experienced. This phenomenon, known as overtraining syndrome (OTS) [45], has been associated with a deterioration in endurance performance [46].

The vascular system is important for the inflammatory response because of the transport of systemic immune cells to the site of inflammation. In vessels, acute and chronic inflammation could damage the arterial wall and lead to ED. The generation of ROS released by immune cells plays a central role in limiting the bioavailability of NO and increasing the formation of peroxynitrite (ONOO^−^), which is a highly unstable ROS involved in vascular inflammation, hypertrophy, fibrosis, and ED [47].

### 1.3. Hesperidin Supplementation: A Potential Ergogenic Aid

The use of ergogenic aids as a strategy to improve exercise performance is widespread among elite as well as recreational athletes [48,49,50,51]. The term “ergogenic aid” includes any training method, mechanic device, or nutritional and pharmacological approach that can improve exercise performance capacity and/or enhance training adaptation [52].

Polyphenols, among other nutritional supplements, have been investigated as ergogenic aids. As their antioxidant and anti-inflammatory role is well-known, polyphenol supplementation could provide an efficient strategy to counteract exercise-related inflammation and prevent cell damage due to an excess of reactive oxygen species (ROS) [53]. Moreover, polyphenols showed the ability to attenuate the delayed onset of muscle soreness (DOMS) [54,55,56,57], a symptom of exercise-induced muscle damage [58]. Other beneficial effects of polyphenols are their capacity to improve physical performance [59] and increase time to exhaustion [60], their anti-fatigue effect [61], and their ability to increase markers of mitochondrial biogenesis (e.g., PGC-1α, SIRT1, mtDNA, and cytochrome *c*) that are associated with maximal endurance capacity [62]. Taken together, polyphenol supplementation can be used as an ergogenic aid to positively impact exercise performance capacity.

Hesperidin (C_28_H_34_O_15_) is a flavanone belonging to the class of flavonoids, one of the most common and widely distributed groups of plant phenolics, which is abundantly present in citrus fruits [63,64]. Orally administered hesperidin (hesperetin-7-O-rutinoside) is converted to the active aglycone hesperetin by an enzyme that is expressed by intestinal microbiota and subsequently absorbed by the gastrointestinal tract [65]. Human studies showed that after the consumption of orange juice, the maximal plasma concentration of hesperidin (0.1–2.2 micromol/L) is reached between 5 and 7 h after ingestion and is still detected in plasma after 10 h [66,67,68,69]. There are indications that hesperidin supplementation has anti-inflammatory [70,71,72], lipid-lowering [73,74,75], neuro-protective [76,77], and insulin-sensitizing properties [78]. Interestingly, hesperidin has also been investigated for its effects on exercise performance. It is essential to measure the level of endogenous antioxidants, endothelial function, and muscle oxygen supply of a person to determine the right dosage of hesperidin supplementation [79].

This review aims to provide an overview of the existing research evidence on hesperidin supplementation as a potential ergogenic aid. The growing interest in the effects of hesperidin on improved human performance is translated into an increasing number of randomized controlled trials (RCTs) performed on athletes. The exact molecular mechanisms through which hesperidin could lead to enhanced exercise performance are not yet clear. Therefore, in this paper, we investigate the potential molecular mechanisms that could provide sufficient scientific evidence regarding its efficacy, namely improved endothelial function and reduced exercise-induced oxidative stress and inflammation. We selected in vivo RCTs, animal studies, and in vitro studies in which hesperidin, its aglycone form hesperetin, hesperetin-metabolites, or orange juice (in which the hesperidin content is known) are supplemented at any dosage. Studies using combined supplements have been excluded from this narrative review.

## 2. Hesperidin Increases Endothelial Function

### 2.1. Hesperidin and Hesperetin Increase NO Production and Decrease Monocyte Adhesion in Endothelial Cells

The effects of hesperidin and hesperetin or their metabolites on endothelial function have been shown in vitro (Table 1). Rizza et al. found that the treatment of bovine aortic endothelial cells (BAECs) with 1μM and 10 μM of hesperetin for 10 min acutely increased cellular levels of phosphorylated (p) 5′AMP-activated protein kinase (AMPK) and protein kinase B (Akt) [71]. Both kinases regulate the activity of endothelial nitric oxide synthase (eNOS), resulting in the increased production of NO. Accordingly, hesperetin treatment of BAECs increased the levels of p-eNOS with a corresponding increase in the NO production [71]. Other studies confirmed the stimulatory effects of hesperidin, hesperetin, or their metabolites with exposure times ranging from 30 min to 24 h on NO production in human umbilical vein endothelial cells (HUVECs) [80,81,82]. The effects of hesperidin on NO production seem to be dose-dependent. Chiou et al. also found decreased levels of strain-induced ET-1 after treatment with hesperidin [82].

In addition to these effects on vasoactive factors, Rizza et al. showed that pre-treatment with hesperetin (10 µM, for 1 h) reduces the TNF-α-stimulated expression of vascular cell adhesion molecule 1 (VCAM-1) as well as TNF-α-stimulated monocyte adhesion [71]. This is in line with other studies showing decreased VCAM-1 levels and decreased monocyte adhesion in TNF-α-stimulated HUVECs pre-treated with hesperetin and its metabolites [83,84]. However, no significant effect was found on the intracellular adhesion molecule 1 (ICAM-1) protein expression [84]. VCAM-1 and ICAM-1 are endothelial adhesion molecules that promote monocyte accumulation in the arterial intima. Increased expression of VCAM-1 was shown to play a major role in the initiation of atherosclerosis [85].

### 2.2. Hesperidin and Hesperetin Decrease Blood Pressure and Increase Endothelium-Dependent Vasodilation in Hypertensive Rats

The effects of hesperidin, hesperetin, and their metabolites on blood pressure and the vasodilatory response were examined in hypertensive rat models (Table 2). Male Sprague–Dawley rats with hypertension showed decreased systolic blood pressure (SBP) and diastolic blood pressure (DBP) when treated with 15 mg/kg and 30 mg/kg of hesperidin for 5 weeks. Furthermore, increased plasma levels of nitric oxide metabolites (NOx) were found [86]. Administration with hesperetin and its metabolite hesperetin-7-O-glucuronide (HPT7G) (but not Hesperetin-3′-O-glucuronide (HPT3′G)) for 3 min resulted in decreased SBP in hypertensive rats, whereas DBP did not change [87]. In the same study, thoracic aortic rings were isolated from spontaneously hypertensive rats (SHRs) and exposed to 100 µM of HPT7G and HPT3′G. HPT7G but not HPT3′G treatment significantly enhanced endothelium-dependent vasodilation but did not alter endothelium-independent vasodilation. In aortic rings from normotensive control rats (Wistar Kyoto rats), the hesperetin metabolites did not change endothelium-dependent and endothelium-independent vasodilation [87].

### 2.3. Hesperidin Increases Flow-Mediated Vasodilation and Decreases sVCAM-1 and sICAM-1 in Humans

RCT studies investigating the effects of hesperidin supplementation on endothelial function are collected in Table 3. ED is characterized by reduced vasodilation, which is non-invasively evaluated in vivo via ultrasound flow-mediated vasodilation (FMD) of the peripheral artery [88]. Since FMD responds rapidly to new drug and bioactive substance therapies, it is considered a good marker to assess endothelial function in interventional trials [88]. ED is also characterized by a pro-inflammatory state, which creates favourable conditions for cytokine secretion by immune cells and an increased expression of adhesion molecules on the endothelial cells of the damaged arterial wall [89]. Through the mechanisms of proteolytic cleavage or alternative splicing, adhesion proteins are released in a circulatory form that can be measured in the plasma [90,91]. The released adhesion molecules are an indicator of ED and the pro-inflammatory state. Hence, the studies collected in this review also evaluated the effects of hesperidin supplementation on endothelial function through the increased serum levels of adhesion molecules such as soluble VCAM-1, soluble ICAM-1, and soluble P-selectin (sP-selectin) [92,93]. Hypertension, also known as high blood pressure, is an important risk factor for ED [93]. Therefore, in the following studies, alterations in SBP and DBP were also assessed. When discussing the results of these studies, a distinction has been made between acute and chronic hesperidin supplementation.

### 2.4. Acute Supplementation

Two studies indicate that blood flow parameters improve 6h after hesperidin supplementation [94,95]. In healthy subjects, the acute administration of 292 mg of hesperidin was able to improve microvascular reactivity measured using combined laser-Doppler flowmetry and iontophoresis [94]. The acute administration of 600 mg of hesperidin significantly improved ischaemic reactive hyperaemia (IRH), a measure of endothelial-dependent vasomotor function, in hypertensive subjects [95]. IRH was measured using a laser-Doppler linear flowmeter taking into account blood perfusion, whereas distal ischaemia was induced by inflating a blood-pressure cuff placed above the elbow to supra-systolic pressure.

Furthermore, supplementation with water-dispersible hesperetin was able to positively impact blood flow in women with cold sensitivity within 70 min after intake [80]. Both concentrations of 17 mg and 170 mg significantly suppressed the drop in blood flow in the air-conditioned room at 22 °C. Schar et al., on the other hand, did not observe any statistically significant changes in multiple vascular function parameters (P-selectin expression, blood pressure, and baroreflex sensitivity) when 320 mg of hesperidin was ingested 5 h before testing in men at moderate risk of cardiovascular disease (CVD) [96]. As noted by the authors, this could be explained by the fact that the plasma concentrations of total flavanone metabolites are only increased until 5 h after hesperidin ingestion.

### 2.5. Chronic Supplementation

FMD significantly improved in three studies evaluating the chronic supplementation of hesperidin: with an oral dosage of 159.5 mg/day of hesperidin for 7 days in adult subjects with increased cardiovascular risk [97] and 500 mg/d for 3 weeks in individuals with metabolic syndrome [71]. Salden et al. induced acute, reversible ED using a high-fat meal in subjects with a baseline FMD ≥3% [98]. In this study, hesperidin supplementation (450 mg/day for 6 weeks) significantly protected against postprandial FMD impairment compared to the placebo. Yari et al. recorded a BP-lowering effect after hesperidin intake; SBP significantly decreased in subjects with metabolic syndrome after hesperidin intake (1 g/day for 12 weeks) compared to the placebo [75]. In the study by Morand et al., DBP was significantly decreased after 4 weeks of hesperidin supplementation (292 mg/day) [94]. The endothelial-dependent vasomotor function marker IRH improved after oral intake of 600 mg/day of hesperidin during an intervention of 12 weeks [95]. In one study, the chronic effect of hesperidin supplementation (292 mg/day for 4 weeks) was evaluated on NO production. Despite no significant change compared to the placebo, an increasing trend in NOx was recorded in the intervention group [94].

A significant decrease in sVCAM-1 and sICAM-1 was observed after 6 weeks of hesperidin supplementation (450 mg/day) compared to the placebo [98]. No significant changes were recorded in the same biomarkers in the studies of Rizza et al. and Morand et al. where the hesperidin supplementation lasted 3 weeks (500 mg/day) and 4 weeks (292 mg/day), respectively [71,94]. Those findings may suggest that a longer supplementation with the flavanone hesperidin is required to significantly affect the serum levels of the abovementioned cellular adhesion molecules.

## 3. Hesperidin Reduces Exercise-Induced Oxidative Stress

### 3.1. Hesperidin and Hesperetin Function as an Antioxidant In Vitro

The results showed that the ROS scavenging activity (with the exception of **·**NO scavenging) of hesperidin/hesperetin was comparable to the mentioned standards (Table 4) [99,100]. Furthermore, hesperetin decreased cellular ROS formation induced by *tert*-butylhydroperoxide (*t*-BHP) and lipopolysaccharides (LPS) in in vitro models using multiple cell types (including endothelial cells, hepatic cells, macrophage cells, and fibroblasts) [82,100,101,102]. Additionally, Kaplana et al. and Chen et al. showed that hesperidin treatment reduced the by-products of lipid peroxidation in the human erythrocyte membrane, measured as thiobarbituric acid-reactive substances (TBARS) and malondialdehyde (MDA), respectively [99,101]. Hesperidin and other polyphenols also showed the potential to affect the endogenous antioxidant status by increasing nuclear factor erythroid 2-related factor 2 (Nrf2) nuclear translocation. In human hepatocytes, increased Nrf2 translocation leads to increased mRNA and protein levels of endogenous antioxidants (e.g., SOD1, GST, thioredoxin, and HO-1) and enhances their activities [101,103].

### 3.2. Hesperidin Decreases ROS and Increases Antioxidant Markers in Rats

Rats were supplemented with hesperidin for a duration ranging from 10 days to 5 weeks (Table 5). In the study of Estruel-Amades et al., Wister rats were trained for five weeks (five days per week) including two exhaustion tests and three trainings per week [104]. The oxidative status was determined before and immediately after an additional exhaustion test. Hesperidin prevented the increase in ROS production by peritoneal macrophages induced by the exhaustion test. Moreover, supplementation with hesperidin avoided the decrease in SOD activity in the thymus and the decrease in CAT activity in the spleen and liver induced by the exhaustion test. Sedentary animals supplemented with hesperidin showed decreased activity of SOD, CAT, and GPx in the mentioned tissue sections. The same applies to the trained animals in which hesperidin supplementation led to either a decrease or no change in antioxidant activity compared to the controls (Table 5) [104]. In the study of El-Sayed et al., the neurotoxin acrylonitrile was used to induce ROS formation in rat brain tissue [105]. Supplementation with hesperidin (200 mg/kg/day) ameliorated the acrylonitrile-induced alterations in brain lipid peroxidation and increased the acrylonitrile-induced reduction in GSH, SOD, CAT, GPx, and glutathione-s-transferase (GST) levels in the brain. Furthermore, increased SOD and GPx levels and decreased CAT levels were found in hesperidin-supplemented rats compared to control rats without any treatment with acrylonitrile. According to this, Sahu et al. showed that hesperidin supplementation with the same dosage for 10 days leads to decreased cisplatin (a cancer treatment known to induce nephrotoxicity)-induced levels of ROS and TBARS and increased activity of antioxidants (including SOD, GSH, CAT, GPx, GST, and glutathione reductase (GR)) in rat kidneys [106]. Without stimulating ROS production, no significant differences in oxidative status were found between hesperidin-treated and control animals [106]. Moreover, a study in hypertensive rats showed decreased vascular superoxide production and decreased plasma levels of MDA after a 5-week administration with hesperidin [86].

### 3.3. Hesperidin Supplementation Increases CAT and Decreases MDA after Strenuous Exercise Performance in Humans

Acute supplementation of hesperidin (500 mg) increased the endogenous antioxidant enzyme catalase (CAT) in venous blood samples after a strenuous exercise performance measured by a Wingate test on a cycle ergometer in male amateur cyclists (Table 6) [107]. On the other hand, the concentration of other endogenous antioxidant markers, such as superoxide dismutase (SOD) and glutathione (GSH), and lipid oxidation markers, such as thiobarbituric acid-reactive substances (TBARS), did not show any significant difference between the intervention and control groups, despite a decreasing trend observed for SOD in the intervention group.

Acute supplementation with 217 mg of hesperidin in healthy soccer players decreased the lipid peroxidation marker malondialdehyde (MDA) post-exercise in plasma [108]. Plasma total antioxidant status (TAS) significantly increased after exercise in both the intervention and placebo groups; however, there was no significant difference present between the control and hesperidin intervention groups.

## 4. Hesperidin Reduces Inflammatory Markers

### 4.1. Hesperidin and Hesperetin Decrease Pro-Inflammatory Responses in LPS-Stimulated Macrophages

The effects of hesperidin/hesperetin on inflammatory responses were investigated in Macrophage RAW264.7 cells (Table 7). Treatment with HPT7G for 24 h showed decreased LPS-induced inflammatory responses measured by a decrease in the mRNA expression of IL-6, IL-1β, TNF-α (only at a concentration of 50μM), and COX-2, and a decreased production of NO, IL-6, and IL-1β. No effects of the flavanone were found on the LPS-induced production of TNF-α [109]. Other studies of the same cell type found that hesperidin and hesperetin exposure with a duration ranging from 30 min to 24 h resulted in a decreased LPS-induced production of PGE_2_, NO, and NO_2_, and decreased protein levels of COX-2 and iNOS [102,110,111]. Furthermore, conflicting results were found concerning the effects of hesperidin and hesperetin on the activation of NF-κB. Although one study found decreased NF-κB activity, the study of Kazlowska et al. showed no effects of the flavanone on NF-κB and iNOS promotor activity [102,111].

### 4.2. Hesperidin Decreases Renal and Plasma Levels of TNF-α in Rat and Mouse Models

The effects of short-term (3 h) and long-term (10 days and 5 weeks) supplementation with hesperidin on TNF-α levels were investigated in animal studies (Table 8). Treatment with 0.3 mg, 1 mg, and 3 mg hesperidin three hours before LPS stimulation led to decreased plasma levels of TNF-α in female mice [112]. The same was observed in male Wistar rats in which 10 days of supplementation with 200 mg/kg/day of hesperidin led to a decrease in the cisplatin (a cancer treatment known to induce nephrotoxicity)-induced increase in renal TNF-α. In the same study, a reduction in cisplatin-induced neutrophil infiltration was observed after supplementation with hesperidin, assessed by the measurement of renal myeloperoxidase (MPO) activity [106]. Moreover, in hypertensive rats, 5 weeks of hesperidin supplementation resulted in decreased plasma values of TNF-α [86].

### 4.3. Hesperidin Decreases CRP, TNF-α, and IL-6 in Humans

In RCTs with hesperidin supplementation for a period of 1–12 weeks performed in healthy adults as well as individuals with medical conditions, such as rheumatoid arthritis, metabolic syndrome (MetS), or increased cardiovascular risk, decreased levels of CRP tumour necrosis factor-alpha (TNF-α) and interleukin-6 (IL-6) were found (Table 9). A significant decrease in CRP, TNF-α, and IL-6 concentrations was measured after 7 days of hesperidin supplementation (159.5 mg/day) in subjects with increased cardiovascular risk [97]. An amount of 1 g of hesperidin per day for 12 weeks decreased TNF-α but not in CRP in subjects with MetS [75]. Kometani et al. recorded a significant decrease in CRP concentration in subjects with arthritis after 12 weeks of supplementation with 3 g of hesperidin per day compared to the placebo [113]. When tested in healthy men, 292 mg hesperidin per day for 4 weeks did not show an effect on IL-6 and CRP concentrations in the intervention group compared to the placebo [94].

In summary, the available human studies indicate that the CRP concentration in serum can be decreased by hesperetin supplementation. Two of these studies also decreased TNF-α levels in serum after hesperidin supplementation compared to controls, whereas the effects of the supplementation on IL-6 levels were inconclusive.

## 5. Hesperidin Improves Exercise Performance

### 5.1. Hesperidin Supplementation Increases Maximum Running Performance in Rats

For a period of five weeks, female rats performed a maximum distance run until exhaustion two times per week and were supplemented with 200 mg/kg of hesperidin or a placebo three times per week. Non-supplemented animals achieved the highest performance in week two, in which they ran about 134% of the maximum distance compared to the first exhaustion test. Animals supplemented with hesperidin showed a significantly better performance compared to the control group, reaching their peak performance in week three, running 158% of the maximum distance compared to the first test (Table 10) [104].

### 5.2. Hesperidin Improves Anaerobic Exercise Performance Outcomes in Human

Ingesting 500 mg of hesperidin 5h before a repeated sprints test (Wingate test) was able to improve anaerobic performance outcomes (average power (W); maximal speed (rpm); and total energy (J)) in the intervention group compared to the placebo [107] (Table 11). Ingesting 217 mg of hesperidin 2.5 h before a Yo-Yo intermittent recovery test (YYIRT) did not result in a significant improvement in the ratings of perceived exertion (RPE) and maximal oxygen uptake (VO_2_max) [108]. However, an increasing trend in VO_2_max was recorded in the intervention group compared to the placebo. VO_2_max is defined as the maximum rate of oxygen consumption measured during severe exercise [114]. In exercise physiology, VO_2_max is used to assess endurance performance and it is limited by the ability of the cardiorespiratory system to deliver oxygen to the exercising muscles [15]. No significant improvement in the estimated VO_2_max was recorded during a 10 min time trial on a cycle ergometer after 4 weeks of hesperidin (450 mg/day) supplementation [115]. In the same study, performance outcomes such as power (W) and VO_2_/power ratio significantly improved in the intervention group compared to the placebo, resulting in a higher amount of power produced per unit of oxygen consumed (VO_2_/power ratio).

Martínez-Noguera et al. and Van Iersel et al. both tested the chronic effects of hesperidin supplementation on sport performance outcomes after a Wingate test [116,117]. Supplementation with 500 mg/day of hesperidin for 8 weeks significantly increased absolute peak power (W) and relative peak power (W) in male amateur cyclists [116]. The oral ingestion of 360 mg and 450 mg of hesperidin/day for 4 weeks significantly improved average power (W) and 5 s peak power (W) recorded during a Wingate anaerobic test performed in trained healthy subjects [117]. Average power (W) was still significantly improved after 8 weeks of hesperidin supplementation (360 mg/day). Moreover, Martínez- Noguera et al. also evaluated the effects of a 500 mg/day hesperidin supplementation for 8 weeks after an incremental test until exhaustion and found a significant improvement in maximum power (W) and estimated functional threshold power (FTP) (W) [116].

## 6. Discussion and Conclusions

**Figure 1:**
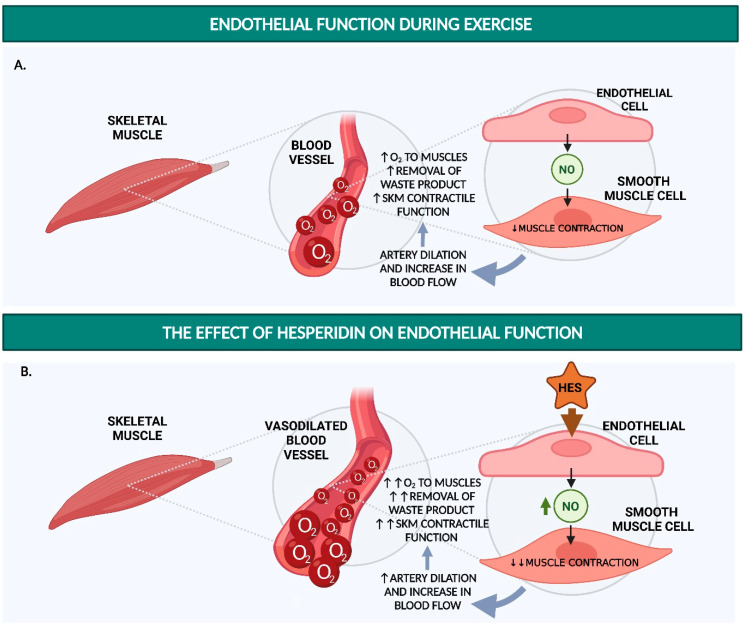
Schematic summary of the potential mechanism of action for the hesperidin effect on endothelial function during exercise. (**A**) During exercise, the release of nitric oxide (NO) by endothelial cells causes the relaxation of the smooth muscle cells, which leads to the dilation of an artery and an increase in blood flow. (**B**) Hesperidin increases the endothelial cells’ NO production. This process leads to higher artery dilation, which further improves blood flow. During exercise, improved skeletal muscle perfusion and the consequent increase in oxygen (O_2_) efflux to the muscle can improve endurance performance. The figure was created with BioRender.com. Abbreviations: HES = hesperidin; NO = nitric oxide; O_2_ = oxygen; SKM = skeletal muscle; Increased: ↑ < ↑↑; Decreased ↓ < ↓↓.

**Figure 2:**
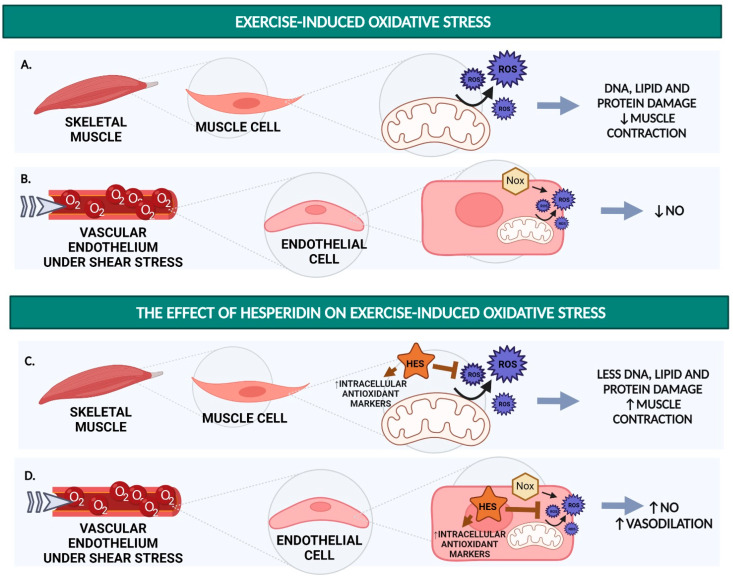
Schematic summary of potential mechanism of action for the hesperidin effect on exercise-induced oxidative stress. (**A**) Contractile activity of skeletal muscle tissue leading to a higher oxygen demand could induce an increased formation of ROS as a result of the excessive mitochondrial activity leading to incomplete oxidative phosphorylation during exercise. In athletes performing extreme endurance exercise, the constant rise in ROS production could lead to damage to DNA, lipids (lipid peroxidation), or protein and attenuation in muscle contraction. (**B**) Increased blood flow (and thereby increased shear stress) during exercise leads to increased endothelial ROS production, which reacts with NO. Increased ROS production by the endothelium leads to decreased NO availability. (**C**) Hesperidin, acting as an antioxidant, helps to prevent the side effects of excessive ROS formation in the muscle cells. Moreover, hesperidin increases endogenous antioxidant enzymes. These two mechanisms combined help prevent cell damage and the decline in muscle contraction signalling pathways leading to stimulation in force production. (**D**) When supplemented with hesperidin, endothelial ROS production will be decreased, preventing the decrease in NO production caused by shear stress. The figure was created with BioRender.com. Abbreviations: HES = hesperidin; NO = nitric oxide; NOX = NADPH oxidase; O_2_ = oxygen; ROS = reactive oxygen species; Increased: ↑; Decreased: ↓.

**Figure 3:**
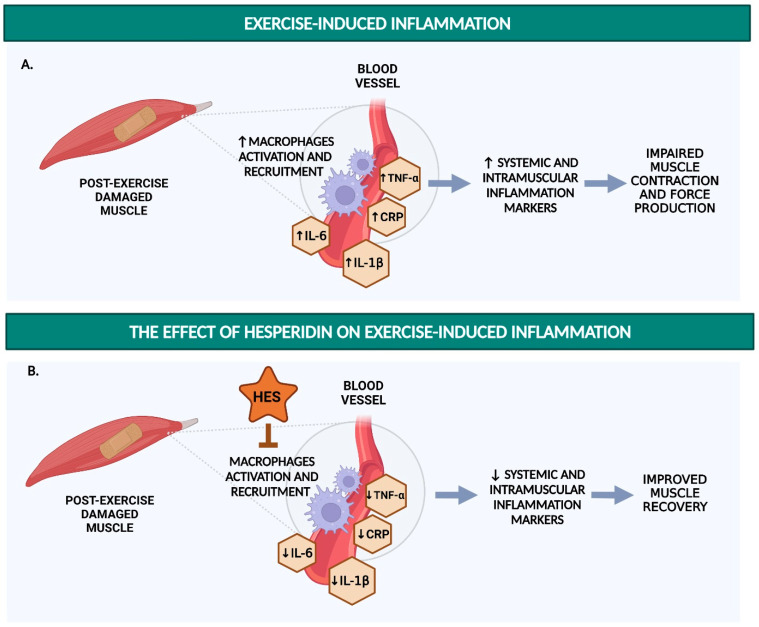
Schematic summary of potential mechanism of action for the hesperidin effect on exercise-induced inflammation. (**A**) Exhaustive exercise leads to macrophage activation, which activates an acute inflammatory response characterized by increases in circulatory and intramuscular inflammatory markers such as C-reactive protein (CRP), cytokines (tumour necrosis factor-alpha (TNF-α), interleukin-6 (IL-6), and interleukin-1beta (IL-1β). Without adequate post-exercise/competition recovery periods, an excessive inflammatory response could lead to impaired muscle contractions and force generation. (**B**) Hesperidin shows the potential to inhibit macrophage activation and recruitment and decrease markers of exercise-induced inflammation, potentially speeding up the recovery process and, therefore, improving exercise performance. The figure was created with BioRender.com. Abbreviations: CRP = C-reactive protein; HES = hesperidin; IL-1β = interleukin-1beta; IL-6 = interleukin-6; TNF-α = tumour necrosis factor-alpha; Increased: ↑; Decreased: ↓.

The studies collected in this review show the potential of hesperidin, hesperetin, and their metabolites to enhance exercise performance by (i) improving endothelial function (via increased NO availability; Figure 1), (ii) reducing oxidative stress (by acting as an antioxidant, e.g., as a ROS scavenger or enhancer of endogenous antioxidant capacity; Figure 2), and (iii) inhibiting the production of pro-inflammatory cytokines to prevent excessive post-exercise inflammation (Figure 3).

In vitro studies investigating the effects of hesperidin, hesperetin, and their metabolites in endothelial cells highlight the potential of the flavanone to enhance the production of NO in the vascular endothelium. There is growing evidence showing that increased NO availability can improve exercise-related performance through enhanced tissue oxygenation (due to blood vessel vasodilation) combined with increased metabolic efficiency in active skeletal muscle [118]. Increased NO availability can enhance skeletal muscle metabolic efficiency by increasing contractile function through alterations in calcium availability and sensitivity in the sarcoplasmic reticulum, resulting in the reduced ATP cost of the muscle force production [119]. Skeletal muscle contraction requires ATP both for the interaction between actin and myosin (actomyosin-ATPase) and for the calcium (Ca^2+^) pumping in the sarcoplasmic reticulum (Ca^2+^-ATPase) [119]. NO, being able to reduce Ca^2+^ release from the sarcoplasmic reticulum [120] and inhibit Ca^2+^-ATPase activity [121], can decrease the energetic cost of muscle force production. This allows high-intensity exercise to be tolerated for a greater period of time. The combination of improved oxygen delivery to the muscle and the related mitochondrial capacity is very important as too much oxygen could induce oxidative stress by overloading the mitochondrial respiration system. Exhaustive aerobic exercise has recently been shown to attenuate maximal skeletal muscle mitochondrial respiratory capacity through the inhibition of oxidative phosphorylation [122]. When it comes to athletes, this likely transient, mitochondrial defect could amplify the exercise-induced development of fatigue [123]. Therefore, investigating the effects of hesperidin on mitochondrial capacity could be an important area for future research.

The studies collected in this review showed enhanced vasodilator responses after supplementation with hesperidin in both healthy and unhealthy individuals/animals. Although in subjects with hypertension, there is a different regulation in blood vessel vasomotor responses compared to healthy people and, therefore, athletes [93,124]. Future studies performed on healthy, trained subjects are needed to assess the efficacy of hesperidin supplementation on vasomotor responses and endothelial function and to eventually translate those effects into improvements in exercise performance.

The included studies were consistent in the ability of hesperidin and hesperetin to inhibit ROS production in a variety of cell types and tissues. Despite the broad amount of literature supporting the role of hesperidin in antioxidant cellular defences, there is still a lack of studies focusing specifically on its effects on skeletal muscles. More RCTs should be conducted to ascertain the effects of hesperidin on oxidative status after exercise [125]. Furthermore, future investigations should assess the baseline levels of endogenous antioxidants in the muscles and endothelium of trained/untrained and healthy/unhealthy subjects. As there could be differences in the baseline antioxidants between individuals, this knowledge could be used to determine the most effective and personalized dose of hesperidin supplementation. Moreover, it is important to highlight the fact that hesperidin works as an exogenous antioxidant and if reacted with ROS, it cannot be converted to its reduced form again by endogenous antioxidant enzymes. Therefore, it is recommended to supplement hesperidin multiple times per day depending on individual needs to ensure the sufficient availability of the reduced form of hesperidin or enhance the endogenous antioxidant network to channel the reactivity of radicals into the antioxidant network [126]. Finally, it would be interesting to investigate whether hesperidin can decrease ROS formation in vessels surrounding the contracting muscles to see if this can be linked to improvements in NO availability and muscle perfusion during exercise.

Hesperidin and hesperetin showed good anti-inflammatory properties by decreasing the production and plasma levels of pro-inflammatory markers. Despite the evidence from studies performed on untrained and unhealthy subjects, we do not have enough data to support the role of hesperidin in restraining systemic inflammation in overtrained subjects. More research is needed to validate our hypothesis that the anti-inflammatory properties of hesperidin can lead to a reduction in intramuscular inflammation and muscle damage, and in this way result in increased exercise performance. Future studies should not only investigate the effects of hesperidin supplementation on systemic post-exercise inflammation markers but also evaluate the changes in intramuscular inflammation markers via skeletal muscle biopsies.

Finally, the effects of hesperidin supplementation on improved exercise performance have been investigated. In rats, supplementation with hesperidin led to increased performance in maximal running distance. In trained athletes, both acute and chronic hesperidin intake was able to improve multiple anaerobic exercise outcomes (e.g., power, speed, energy). Further studies are needed to assess the effects of hesperidin supplementation on endurance exercise in humans.

In conclusion, the ergogenic effects that hesperidin can bring to the spectrum of improved exercise performance are promising and should be investigated further. For elite and recreational athletes, hesperidin could be a promising food supplement to optimize the oxygen and nutrient supplies of the muscles, stimulate muscle contraction, and enhance muscle recovery between training sessions. During exercise, hesperidin supplementation can increase endothelial function, thereby contributing to increased skeletal muscle perfusion and increasing oxygen (O_2_) efflux to the muscle, which is associated with increased endurance performance. Moreover, hesperidin can decrease ROS-mediated damage in muscle cells, which enhances muscle function. Finally, hesperidin can decrease post-exercise-induced inflammation, which potentially speeds up the recovery process and can thereby improve exercise performance. In this way, personalized supplementation with hesperidin seems to increase anaerobic exercise performance, although further research is necessary to draw conclusions regarding the efficiency of hesperidin supplementation for endurance athletes.

## Figures and Tables

**Table 1:** Studies investigating the effects of hesperidin, hesperetin, and their metabolites on endothelial function markers in studies performed in vitro.

Author, Year, Country	Cell Type	Treatment Characteristics	Treatment Duration	Endothelial Function Outcomes (Hesperidin or Hesperetin vs. Control)
**Rizza et al.** **[71]** **2011** **Italy**	BAEC	Hesperetin 0.01 μM, 0.1 μM, 1 μM, 10 μM	10 min	↑pAMPK protein levels (1 μM, 10 μM) ↑pAkt protein levels (1 μM, 10 μM) ↑p-eNOS protein levels (1 μM, 10 μM) =pAMPK, pAkt and p-eNOS protein levels (0.01 μM, 0.1 μM)
			1 h	↑NO production (10 μΜ) =NO production (0.01 μM, 0.1 μM, 1 μM)
				↓TNF-α-stimulated VCAM-1 protein levels (10 μM) ↓TNF-α-stimulated monocyte adhesion (10 μM)
**Takumi et al.** **[80]** **2012** **Japan**	HUVECs	Hesperetin, HPT7G 25 µM, 50 µM	24 h	↑Release of NO, in a dose-dependent manner
**Liu et al.** **[81]** **2008** **China**	HUVECs	Hesperetin 12.5 μM, 25 μM, 50 μM, 100 μM	24 h	↑Release of NO in a dose-dependent manner
				↑eNOS mRNA expression (50 μM) ↑eNOS protein levels (50 μM)
**Chiou et al.** **[82]** **2008** **Taiwan**	HUVECs	Hesperidin 1 µM, 10 µM, 100 µM	30 min prior to strain treatment (computer-controlled application of sinusoidal negative pressure)	↓strain-induced ET-1 secretion (10 µM, 100 µM) =strain-induced ET-1 secretion (1 µM)
			30 min	↑NO production (100 µM) ↑eNOS phosphorylation (100 µM) ↑Akt phosphorylation (100 µM)
			60 min	↑NO production (10 µM, 100 µM) =NO production (1 µM) ↑NOS activity (10 µM, 100 µM) =NOS activity (1 µM) ↑eNOS phosphorylation (100 µM) =Akt phosphorylation (100 µM)
**Chanet et al.** **[83]** **2013** **France**	HUVECs	Hesperetin, HPT3′G, HPT3′S, HPT7G 2 μM	24 h	↓TNF-α-stimulated monocyte adhesion
**Nizamutdinova et al.** **[84]** **2008** **Korea**	HUVECs	Hesperidin, hesperidin methyl chalone 1 µM, 5 µM, 10 µM, 50 µM	24 h	↓TNF-α-stimulated VCAM-1 protein expression (5 µM, 10 µM, 50 µM) =TNF-α-stimulated VCAM-1 protein expression (1 µM) =TNF-α-stimulated ICAM-1 protein expression (1 µM, 5 µM, 10 µM, 50 µM)
				↓TNF-α-stimulated monocyte adhesion (5 µM, 10 µM, 50 µM) ↓TNF-α-stimulated monocyte adhesion (1 µM)

↑: statistically significant increase; ↓: statistically significant decrease; = no significant change. Abbreviations: BAEC = bovine aortic endothelial cells; HUVECs = human umbilical vein endothelial cells; (p)AMPK = (phosphorylated) 5′AMP-activated protein kinase; (p)Akt = (phosphorylated) protein kinase B; (p)-Enos = (phosphorylated) endothelial nitric oxide synthase; NO = nitric oxide; TNF-α = tumour necrosis factor-α; VCAM-1 = vascular cell adhesion molecule 1; ICAM-1 = intracellular adhesion molecule 1; HPT7G = hesperetin-7-O-glucuronide; HPTG’3 = hesperetin-3′-O-glucuronide; HPT′3S = hesperetin-3′-O-sulphate; ET-1 = endothelin-1.

**Table 2:** Studies investigating the effects of hesperidin, hesperetin, or metabolites on endothelial function markers in animal studies.

Author, Year, Country	Sample Characteristics	Intervention Characteristics	Intervention Duration	Endothelial Function Outcomes (Hesperidin or Hesperetin vs. Control Groups)
**Maneesai et al.** **[86]** **2018** **Thailand**	Male Sprague–Dawley rats with hypertension (treated with L-NAME)	Hesperidin, 15 mg/kg/day and 30 mg/kg/day	5 weeks	↓SBP, DBP ↑plasma NOx
**Yamamoto et al.** **[87]** **2013** **Japan**	Male SHRs	Hesperetin, HPT7G, HPT3′G 5 mg/kg	3 min	↓SBP (hesperetin, HPT7G) =SBP (HPT3′G) =DBP (hesperetin, HPT7G, HPT3′G)
**Yamamoto et al.** **[87]** **2013** **Japan**	Thoracic aortic rings from SHRs and WKY rats	HPT7G HPT3′G 100 µM	20 min	**SHRs:** ↑ACh-induced endothelium-dependent vasodilation (HPT7G) =Ach-induced endothelium-dependent vasodilation (HPT3′G) =SNP-induced endothelium-independent vasodilation (HPT7G, HPT3′G) **WKY:** =ACh-induced endothelium-dependent vasodilation (HPT7G, HPT3′G) =SNP-induced endothelium-independent vasodilation (HPT7G, HPT3′G)

↑: statistically significant increase; ↓: statistically significant decrease; = no significant change. Abbreviations: L-NAME = N^ω^-nitro L-arginine methyl ester; SBP = systolic blood pressure; DBP = diastolic blood pressure; Nox = nitric oxide metabolites; SHRs = spontaneously hypertensive rats; HPT7G = hesperetin-7-O-glucuronide; HPTG’3 = hesperetin-3′-O-glucuronide; WKY = Wistar Kyoto; Ach = acetylcholine; SNP = sodium nitroprusside.

**Table 3:** Studies investigating the effects of hesperidin on endothelial function markers in human studies.

Author, Year, Country	Sample Characteristics (Study Design)	Intervention Characteristics	Intervention Duration	Endothelial Function Outcomes (Hesperidin vs. Control Groups)
**Morand et al.** **[94]** **2011** **France**	n = 24 healthy males Age = 56 (1) y BMI = 27.4 (0.3) kg/m^2^ (RCT)	292 mg hesperidin/day	Acute (6 h before test)	*↑*microvascular reactivity
			Chronic (4 weeks)	↓DBP =sICAM-1 =sVCAM-1 =NOx, *trend for improvement*
**Valls et al.** **[95]** **2021** **Spain**	n = 159 subjects with pre- or stage 1 hypertension Age = 19–67 y BMI = 18.5–40.5 kg/m^2^ (RTC)	600 mg hesperidin/day	Acute (6 h before test)	↑IRH
			Chronic (12 weeks)	↑IRH
**Takumi et al.** **[80]** **2011** **Japan**	n = 10 healthy female subjects Age = 18–22 y (RTC)	17 mg or 170 mg hesperidin	Acute (test within 70 min after intake)	↓drop in blood flow *Comment: while subjects stayed in an air-conditioned room; significant drop in both INT dosages*
**Schar et al.** **[96]** **2015** **UK**	n = 16 men at moderate CVD riskAge = 60.6 (8.4) y BMI = 25.6 (0.8) kg/m^2^ (RCT)	320 mg hesperidin	Acute (5 h before test)	=P-selectin expression = BP =Cardiac BRS
**Buscemi et al.** **[97]** **2012** **Italy**	n = 21 with increased cardiovascular risk Age = 19–67 y BMI = 18.5–40.5 kg/m^2^ (RCT)	159.5 mg/day hesperidin	Chronic (7 days)	↑FMD
**Rizza et al.** **[71]** **2011** **Italy**	n = 24 with MetS Age = 52 (2) BMI = 34.7 (1.5) kg/m^2^ (RCT)	500 mg/day hesperidin	Chronic (3 weeks)	↑FMD =VCAM-1
**Salden et al.** **[98]** **2016** **The Netherlands**	n = 48 subjects with baseline FMD ≥3% Age = 53 (14) y BMI = 29 (2.6) kg/m^2^ (RTC)	450 mg/day hesperidin	Chronic (6 weeks)	↑FMD ↓sVCAM-1 ↓sICAM-1
**Yari et al.** **[75]** **2020** **Iran**	n = 49 subjects with MetSAge = 45.1 (11.1) y BMI = 31.3 (4.9) kg/m^2^ (RCT)	1 g/day hesperidin	Chronic (12 weeks)	↓SBP

↑: statistically significant increase; ↓: statistically significant decrease; = no significant change; data are presented as mean ± SD or as a range. Abbreviations: Aus = arbitrary units (log); BMI = body max index (kg/m^2^); BRS = baroreflex sensitivity; BP = blood pressure; CON = control; DBP = diastolic blood pressure; FDM = flow-mediated dilation; INT = intervention; IRH = ischaemic reactive hyperaemia; MetS = metabolic syndrome; Nox = nitric oxide metabolites; RCT = randomized controlled trial; SBP = systolic blood pressure; sICAM-1 = soluble intercellular adhesion molecule 1; sVCAM-1 = soluble vascular cell adhesion molecule 1.

**Table 4:** Studies investigating the effects of hesperidin, hesperetin and their metabolites on oxidative stress markers in studies performed in vitro.

Author, Year, Country	Cell Type	Radical Scavenging Activity Assay	Treatment Characteristics	Treatment Duration	Oxidative Stress Outcomes (Hesperidin or Hesperetin vs. Control)
**Kalpana et al. 2009** **[99]** **India**	Human erythrocytes	*·*OH, *·*O_2_, *·*NO and ABTS•+ radical scavenging activity assay	Hesperidin, 0.5 mM, 1 mM, 1.5 mM, 2 mM, 2.5 mM Hesperidin 0.5 mM, 1 mM, 1.5 mM, 2 mM, 2.5 mM	Assay-dependent	=free radical scavenging activity compared to ascorbic acid and trolox, in a dose-dependent manner
				30 min	↓H_2_O_2_-induced TBARS production, in a dose-dependent manner
**Kim et al.** **[100]** **2004** **South Korea**	YPEN-1 prostatic endothelial cells	ONOO^−^, *·*O_2_^−^, *·*NO scavenging activity assay	Hesperetin 5 µM, 15 µM, 50 µM, 200 µM	2 h	=ONOO^−^ and ·O2^−^ scavenging activity compared to penicillamine and Trolox, respectively ↓·NO scavenging activity compared to carboxy-PTIO ↓*t*-BHP-induced intracellular ROS generation in a dose-dependent manner
**Chiou et al.** **[82]** **2008** **Taiwan**	HUVECs		Hesperidin, 1 µM, 10 µM, 100 µM	1 h exposure in the presence of strain treatment (computer-controlled application of sinusoidal negative pressure)	= strain-increased ROS formation (1 µM) ↓strain-increased ROS formation (10 µM, 100 µM)
**Chen et al.** **[101]** **2010** **China**	L02 hepatic cells		Hesperidin 20 µM, 40 µM, 80 µM	24 h	=*t*-BHP-induced intracellular ROS levels (20 µM) ↓*t*-BHP-induced intracellular ROS levels (40 µM, 80 µM) =*t*-BHP-induced MDA production (20 µM)↓*t*-BHP-induced MDA production (40 µM, 80 µM)
**Yang et al.** **[102]** **2012** **Taiwan**	Macrophage RAW264.7 cells and fibroblast A7r5 cells		Hesperetin, Hesperetin metabolites extracted from rat serum 1 μM, 5 μM, 10 μM	60 min for RAW264.7 cells 5 min for A7r5 cells	↓LPS-induced intracellular ROS level (1 μM, 5 μM, 10 μM) Hesperetin metabolites showed greater antioxidant potential compared to hesperetin

↓: statistically significant decrease; = no significant change; Abbreviations: HUVECs = human umbilical vein endothelial cells; ROS = reactive oxygen species; ABTS = 2,2′-azino-bis(3-ethylbenzothiazoline-6-sulfonic acid; H_2_O_2_ = hydrogen peroxide; TBARS = thiobarbituric acid-reactive substances; *·*OH = hydroxyl radical; ONOO^−^ = peroxynitrite;*·*O_2_^−^ = superoxide anion;*·*NO = nitric oxide; *t*-BHP = tert-butylhydroperoxide; MDA = malondialdehyde.

**Table 5:** Studies investigating the effects of hesperidin on oxidative stress markers in animal studies.

Author, Year, Country	Sample Characteristics	Intervention Characteristics	Intervention Duration	Oxidative Stress Outcomes (Hesperidin vs. Control Groups)
**Estruel-Amades et al.** **[104]** **2019** **Spain**	Groups of Female Wister rats: Sedentary rats (SED) 5-week-trained rats (T) 5-week-trained rats undergoing an additional exhaustion test (TE)	200 mg/kg of hesperidin three times per week	5 weeks	↓ROS production by peritoneal macrophages induced by the exhaustion test **In thymus tissue:** =CAT activity in all groupsHesperidin prevented the ↓ in SOD activity induced by the exhaustion test ↓SOD activity in SED group **In spleen tissue:** Hesperidin prevented the ↓ in CAT activity induced by the exhaustion test ↓SOD activity in SED and TE groups =SOD activity in T group **In liver tissue:** Hesperidin prevented the ↓ in CAT activity induced by the exhaustion test ↓CAT activity in SED group = CAT activity in T and TE groups ↓SOD activity all groups ↓GPx activity in SED and TE groups =GPx activity in T group
**El-Sayed et al.** **[105]** **2008** **Egypt**	Brain tissue from male Swiss albino rats	Hesperidin 200 mg/kg/day	28 days	=MDA content ↓Acrylonitrile-induced increase in MDA content =GSH, GST content ↑SOD, GPx levels ↓CAT levels ↑Acrylonitrile-induced decrease in GSH, SOD, CAT, GPx, GST levels
**Sahu et al.** **[106]** **2013** **India**	Kidney tissue from male Wistar rats	Hesperidin 100 mg/kg/day, 200 mg/kg/day	10 days	=ROS levels ↓cisplatin-induced increase in ROS (100, 200 mg/kg/day) =TBARS levels ↓cisplatin-induced increase in TBARS (100, 200 mg/kg/day) =SOD, GSH, CAT, GPx, GR, GST activity =cisplatin-induced decrease in GSH, CAT, GPx, GR activity (100 mg/kg/day) ↑cisplatin-induced decrease in SOD, GST activity (100 mg/kg/day) ↑cisplatin-induced decrease in SOD, GSH, CAT, GPx, GR, GST activity (200 mg/kg/day)
**Maneesai et al.** **[86]** **2018** **Thailand**	Male Sprague–Dawley rats with hypertension (treated with L-NAME)	Hesperidin 15 mg/kg/day, 30 mg/kg/day	5 weeks	↓vascular superoxide production (15, 30 mg/kg/day) ↓plasma MDA (15, 30 mg/kg/day)

↑: statistically significant increase; ↓: statistically significant decrease; = no significant change; Abbreviations: L-NAME = N^ω^-nitro L-arginine methyl ester; MDA = malondialdehyde; ROS = reactive oxygen species; CAT = catalase; SOD = superoxide dismutase; GPx = glutathione peroxidase; GSH = glutathione; GST = glutathione S-transferase; TBARS = thiobarbituric acid-reactive substances; GR = glutathione reductase.

**Table 6:** Studies investigating the effects of hesperidin on oxidative stress following physical exercise.

Author, Year, Country	Sample Characteristics (Study Design)	Intervention Characteristics	Intervention Duration	Exercise Test	Exercise-Induced Oxidative Stress Outcomes (Hesperidin vs. Control Groups)
**Martínez-Noguera et al.** **[107]** **2019** **Spain**	n = 15 male amateur cyclists Age = 18–55 y, BMI = 19–25.5 kg/m^2^ (RCT)	500 mg hesperidin	Acute (5 h before exercise)	Repeated sprints test (Wingate test)	=TBARS ↑CAT =SOD =GSH
**Boussetta et al.** **[108]** **2019** **Tunisia**	n = 11 healthy soccer players Age = 22.4 ± 0.5 BMI = 23.2 ± 0.4 kg/m^2^ (RCT)	INT: 217 mg hesperidin CON: placebo	Acute (2.5 h before the test)	Yo-Yo Intermittent Recovery Test (YYIRT)	=TAS ↓MDA

↑: statistically significant increase; ↓: statistically significant decrease; = no significant change; Abbreviations: BMI = body max index; CAT = catalase; CON = control; GSH = glutathione; INT = intervention; MDA = malondialdehyde; MET = metabolic equivalent; RCT = randomized controlled trial; SOD = Superoxide dismutase; TAS = total antioxidant status; TBARS = thiobarbituric acid-reactive substances; Data are presented as mean ± SD or as a range.

**Table 7:** Studies investigating the effects of hesperidin and hesperetin on inflammatory markers in studies performed in vitro.

Author, Year, Country	Cell Type	Treatment Characteristics	Treatment Duration	Inflammatory Outcomes (Hesperidin or Hesperetin vs. Control)
**Shen et al.** **[109]** **2019** **China**	Macrophage RAW264.7 cells	HPT7G 3.13, 6.25, 12.5, 25, 50, 100 and 200 μg/mL	24 h 12 h (for measurement of mRNA expression)	=LPS-induced NO production (3.13, 6.25 µg/mL) ↓LPS-induced NO production (12.5, 25, 50 µg/mL) ↓LPS-induced IL-6 production (50, 100, 200 µg/mL) =LPS-induced IL-6 mRNA expression (50 µg/mL) ↓LPS-induced IL-6 mRNA expression (100, 200 µg/mL) ↓LPS-induced IL-1β production (50, 100, 200 µg/mL) ↓LPS-induced IL-1β mRNA expression (50, 100, 200 µg/mL) =LPS-induced TNF-α production (50, 100, 200 µg/mL) =LPS-induced TNF-α mRNA expression (100, 200 µg/mL) ↓LPS-induced TNF-α mRNA expression (50 µg/mL) =LPS-induced COX-2 mRNA expression (50 µg/mL) ↓LPS-induced COX-2 mRNA expression (100, 200 µg/mL)
**Yang et al.** **[102]** **2012** **Taiwan**	Macrophage RAW264.7 cells and fibroblast A7r5 cells	Hesperetin, Hesperetin metabolites extracted from rat serum 1 μM, 5 μM, 10 μM	18 h exposure for RAW264.7 cells 8 h exposure for A7r5 cells	↓LPS-induced PGE2 production (1 μM, 5 μM, 10 μM in both cell types) ↓LPS-induced COX-2 protein levels (1 μM, 5 μM, 10 μM in both cell types) ↓LPS-induced NO production (1 μM, 5 μM, 10 μM in RAW264.7 cells) =LPS-induced NO production (1 μM, 5 μM, 10 μM in A7r5 cells) ↓iNOS protein levels ((1 μM, 5 μM, 10 μM in both cell types) ↓LPS-induced NF-κB transcriptional activation (1 μM, 5 μM, 10 μM in RAW264.7 cells) Hesperetin metabolites showed greater anti-inflammatory potential compared to hesperetin
**Sakata et al. 2003** **[110]** **Japan**	Macrophage RAW264.7 cells	Hesperidin 10 μM, 20 μM, 30 μM	30 min	=LPS-induced PGE_2_ production (10 μM) ↓LPS-induced PGE_2_ production (20 μM, 30 μM) =LPS-induced COX-2 protein level ((10 μM, 20 μM, 30 μM)) ↓LPS-induced NO_2_ production (10 μM, 20 μM, 30 μM) ↓LPS-induced iNOS protein level (10 μM, 20 μM, 30 μM)
**Kazlowska et al.** **[111]** **2010** **Taiwan**	Macrophage RAW264.7 cells	Hesperidin 5 μg/mL, 15 μg/mL, 80 μg/mL, 125 μg/mL, 150 μg/mL 250 μg/mL	24 h	=LPS-induced NO production (5 μg/mL) ↓LPS-induced NO production (15 μg/mL, 125 μg/mL, 250 μg/mL) =LPS-induced iNOS promoter activity (80 μg/mL, 150 μg/mL, 250 μg/mL) =LPS-induced NF-κB activity (80 μg/mL, 150 μg/mL, 250 μg/mL)

↓: statistically significant decrease; = no significant change; Abbreviations: HPT7G = hesperetin-7-*O-*glucopyranoside; LPS = lipopolysaccharides; NO = nitric oxide; IL-6 = interleukin-6; IL-1β = interleukin-1beta; TNF-α = tumour necrosis factor-alpha; COX-2 = cyclo-oxygenase 2; PGE_2_ = prostaglandin E2; NO_2_ = nitrogen dioxide; iNOS = nitric oxide synthase; NF-κB = nuclear factor kappa-light-chain-enhancer of activated B cells.

**Table 8:** Studies investigating the effects of hesperidin on inflammatory markers in animal studies.

Author, Year, Country	Sample Characteristics	Intervention Characteristics	Intervention Duration	Inflammatory Outcomes (Hesperidin vs. Control Groups)
**Kawaguchi et al.** **[112]** **2004** **Japan**	Female BALB/c and C57L/6 mice	Hesperidin, 0.1 mg, 0.3 mg, 1 mg, 3 mg/mouse	3 h before LPS treatment	↓LPS-induced increase in plasma TNF-α (0.3 mg, 1 mg, 3 mg/mouse) =LPS-induced increase in plasma TNF-α (0.1 mg/mouse)
**Sahu et al.** **[106]** **2013** **India**	Male Wistar rats	Hesperidin 100 mg/kg/day, 200 mg/kg/day	10 days	=renal TNF-α (200 mg/kg/day) =cisplatin-induced increase in renal TNF-α (100 mg/kg/day) ↓cisplatin-induced increase in renal TNF-α (200 mg/kg/day) =renal myeloperoxidase (200 mg/kg/day) ↓cisplatin-induced increase in renal myeloperoxidase (100, 200 mg/kg/day)
**Maneesai et al.** **[86]** **2018** **Thailand**	Male Sprague–Dawley rats with hypertension (treated with L-NAME)	Hesperidin 15 mg/kg/day and 30 mg/kg/day	5 weeks	↓plasma TNF-α (15, 30 mg/kg/day)

↓: statistically significant decrease; = no significant change; Abbreviations: LPS = lipopolysaccharides; TNF-α = tumour necrosis factor-alpha; L-NAME = N^ω^-nitro L-arginine methyl ester.

**Table 9:** Studies investigating the effects of hesperidin on inflammatory markers in human studies.

Author, Year, Country	Subject Characteristics (Study Design)	Intervention Characteristics	Intervention Duration	Inflammatory Outcomes (Hesperidin vs. Control Groups)
**Buscemi et al.** **[97]** **2012** **Italy**	n = 21 subjects with increased cardiovascular risk Age = 19–67 y BMI = 18.5–40.5 kg/m^2^ (RCT)	159.5 mg/day hesperidin	7 days	↓hs-CRP ↓IL-6 ↓TNF-α
**Yari et al.** **[75]** **2020** **Iran**	n = 49 subjects with MetS Age = 45.1 ± 11.1 y BMI = 31.3 ± 4.9 kg/m^2^ (RCT)	1 g/day hesperidin	12 weeks	↓TNF-α =hs-CRP
**Kometani et al.** **[113]** **2008** **Japan**	n = 19 subjects with arthritis Age = 26–49 y (RCT)	3 g/day hesperidin	12 weeks	↓CRP
**Morand et al.** **[94]** **2011** **France**	n = 24 healthy males Age = 56 ± 1 y BMI = 27.4 ± 0.3 kg/m^2^ (RCT)	292 mg/day hesperidin	4 weeks	=CRP =IL-6

↓: statistically significant decrease; = no significant change; Abbreviations: BMI = body max index (kg/m^2^); CON = control; CRP = C-reactive protein hs-CRP = high-sensitivity C-reactive protein; IL-6 = interleukin-6; INT = intervention; MetS = metabolic syndrome; RCT = randomized controlled trial; TNF-α = tumour necrosis factor-alpha. Data are presented as mean ± SD or as a range.

**Table 10:** Studies investigating the effects of hesperidin on exercise performance outcomes in animal studies.

Author, Year, Country	Sample Characteristics (Study Design)	Intervention Characteristics	Intervention Duration	Exercise Test	Exercise Performance Outcomes (Hesperidin vs. Control Groups)
**Estruel-Amades et al.** **[104]** **2019** **Spain**	Female Wistar rats	200 mg/kg of hesperidin three times per week	Chronic (5 weeks)	Maximum distance run until exhaustion test (2 times per week for 5 weeks)	↑ maximum distance during all performed tests (week 1–5)

↑: statistically significant increase.

**Table 11:** Studies investigating the effects of hesperidin on exercise performance outcomes in human studies.

Author, Year, Country	Sample Characteristics (Study Design)	Intervention Characteristics	Intervention Duration	Exercise Test	Exercise Performance Outcomes (Hesperidin vs. Control Groups)
**Martínez-Noguera et al.** **[107]** **2019** **Spain**	n = 15 male amateur cyclists Age = 18–55 y, BMI = 19–25.5 kg/m^2^ (RCT)	500 mg hesperidin	Acute (5 h before exercise)	Repeated sprints test (Wingate test)	↑Average power ↑Maximal speed ↑Total energy
**Boussetta et al.** **[108]** **2019** **Tunisia**	n = 11 healthy soccer players Age = 22.4 ± 0.5 y BMI = 23.2 ± 0.4 kg/m^2^ (RCT)	217 mg hesperidin	Acute 2.5 h before the test)	Yo-Yo intermittent recovery test (YYIRT)	=VO_2_max (increasing trend) =PRE
**Overdevest et al.** **[115]** **2018** **The Netherlands**	n = 39 trained males Age = 18–25 y BMI = 22.1 (0.30) kg/m^2^ (RCT)	500 mg/day citrus fruit extract (450 mg hesperidin/day)	Chronic (4 weeks)	10 min time-trial on a cycle ergometer	↑Δ Power ↓VO_2_/Power ratio = Es VO_2_max
**Martínez-Noguera et al.** **[116]** **2020** **Spain**	n = 40 male amateur cyclists Age = 18–55 y, BMI = 19–25.5 kg/m^2^ (RCT)	500 mg/day hesperidin	Chronic (8 weeks)	Repeated sprints test (Wingate test)	↑Absolute peak power ↑Relative peak power
				Incremental test until exhaustion	↑ Maximum power ↑ Estimated FTP
**Van Iersel et al.** **[117]** **2021** **The Netherlands**	n = 92 moderately trained healthy subjects Age = 24 ± 5 y BMI = 22.4 ± 2.2 kg/m^2^ (RCT)	360 mg or 450 mg hesperidin	Chronic (4 and 8 weeks)	Wingate anaerobic test	↑Average power (360 mg after 4 weeks) ↑Average power (360 mg after 8 weeks) ↑Average power (450 mg after 4 weeks) ↑5 s Peak power (360 mg after 4 weeks)

↑: statistically significant increase; ↓: statistically significant decrease; = no significant change; Abbreviations: BMI = body max index; CON = control; Es VO_2_max = Estimated VO_2_max; FTP = functional threshold power; INT = intervention; MET = metabolic equivalent; RCT = randomized controlled trial; RPE = Ratings of Perceived Exertion; VO_2_max = maximal oxygen uptake; Data are presented as mean ± SD or as a range.
